# Anti-Inflammatory Activities of *Arnica montana* Planta Tota versus Flower Extracts: Analytical, *In Vitro* and *In Vivo* Mouse Paw Oedema Model Studies

**DOI:** 10.3390/plants12061348

**Published:** 2023-03-16

**Authors:** Johann Röhrl, Maria-Riera Piqué-Borràs, Manuela Jaklin, Markus Werner, Oliver Werz, Heinke Josef, Hubert Hölz, Aldo Ammendola, Gerald Künstle

**Affiliations:** 1 Preclinical Development, Weleda AG, 4144 Arlesheim, Switzerland; 2 Department of Pharmaceutical/Medicinal Chemistry, Institute of Pharmacy, Friedrich Schiller University Jena, 07743 Jena, Germany; 3 Quality Control, Weleda AG, 73525 Schwäbisch Gmünd, Germany; 4 Research and Development, Weleda AG, 4144 Arlesheim, Switzerland

**Keywords:** *Arnica montana* planta tota, *Arnica montana* flos, inflammation, NF-κB, leukotrienes, prostaglandins, arachidonic acid, carrageenan-induced paw oedema, 5-lipoxygenase, cyclooxygenase-2

## Abstract

*Arnica montana* is well known for its anti-inflammatory properties. While the anti-inflammatory activity of Arnica flowers (Arnicae flos) has been extensively studied, that of the whole plant (Arnicae planta tota) is less characterized. We compared the ability of Arnicae planta tota and Arnicae flos extracts to inhibit the pro-inflammatory NF-κB—eicosanoid pathway, using several *in vitro* and *in vivo* assays. We showed that Arnicae planta tota inhibited NF-κB reporter activation, with an IC_50_ of 15.4 μg/mL (vs. 52.5 μg/mL for Arnicae flos). Arnicae planta tota also inhibited LPS-induced expression of ALOX5 and PTGS2 genes in human differentiated macrophages. ALOX5 and PTGS2 encode the 5-lipoxygenase (5-LO) and cyclooxygenase-2 (COX-2) enzymes that initialize the conversion of arachidonic acid into leukotrienes and prostaglandins, respectively. Arnicae planta tota inhibited 5-LO and COX-2 enzymatic activity *in vitro* and in human primary peripheral blood cells, with lower IC_50_ compared to Arnicae flos. Finally, Arnicae planta tota applied topically reduced carrageenan-induced mouse paw oedema more efficiently than Arnicae flos. Altogether, Arnicae planta tota displayed a superior anti-inflammatory activity compared to Arnicae flos, suggesting that Arnicae-planta-tota-containing products might be more effective in alleviating the manifestations of acute inflammation than those based on Arnicae flos alone.

## 1. Introduction

*Arnica montana* has been used for several hundred years for the treatment of various ailments, including contusion, wounds, rheumatism and inflammation [1]. Numerous studies have shown the great medicinal value of Arnica flowers (Arnicae flos), most often applied as tincture or ointment on affected skin areas [1,2,3,4,5,6]. *A. montana* tincture is mainly produced from Arnicae flos by ethanolic extraction. The tincture is desiccated by evaporation, and the extract (3–30%) is incorporated into a variety of herbal preparations or pharmaceutical products [1].

*A. montana* contains a high number of biologically active substances, including sesquiterpene lactones, flavonoids and phenolic acids [1,5,6,7]. While sesquiterpene lactones mediate the anti-inflammatory properties of *A. montana*, flavonoids and phenolic acids exert significant antioxidant and antimicrobial activities [1,2,3,5,8,9]. The content of bioactive substances varies in different parts of the plant, but also with geographic location, climate, altitude and maturity of flower head (from bud to withered flower) [1,6,7]. Flower heads are particularly rich in sesquiterpene lactones, such as helenalin (up to 1% dry weight) [1,2], while roots contain high levels of thymol, known for its antioxidant, antimicrobial and anti-inflammatory properties [1,7,10,11,12,13,14,15].

The anti-inflammatory activity of Arnicae flos, notably its ability to inhibit NF-κB signaling and its downstream 5-lipoxygenase (5-LO)- and cyclooxygenase-2 (COX-2)-mediated pathways, is well documented [1,2,8,9,16]. NF-κB is an important mediator of immune response and inflammation [17]. It regulates the transcription of proinflammatory genes such as arachidonate 5-lipoxygenase (ALOX5) and prostaglandin-endoperoxide synthase 2 (PTGS2). ALOX5 and PTGS2 encode 5-LO and COX-2, which are key enzymes in the biosynthesis of eicosanoids, namely leukotrienes and prostaglandins, respectively, thereby promoting inflammation, pain and fever [16,18]. Both leukotrienes and prostaglandins are directly involved in the processes leading to the early signs of inflammation, including redness, swelling (oedema) and pain [16,18,19,20,21,22,23,24]. While most studies have investigated the anti-inflammatory activities of Arnicae flos, very little is known of the anti-inflammatory properties of Arnicae planta tota [1,25,26]. We hypothesized that, due to an increased and broader content of bioactive substances, Arnicae planta tota extracts might exert greater anti-inflammatory activities than Arnicae flos extracts.

In this study, we compared the ability of Arnicae planta tota and Arnicae flos to inhibit the NF-κB signaling pathway and its messengers (5-LO, COX-2), using *in vitro* enzymatic assays, cell-based assays and an *in vivo* mouse model of inflammation. We showed that Arnicae planta tota presented superior anti-inflammatory activity in these assays, compared to Arnicae flos. Our data suggest that products containing Arnicae planta tota might be more effective in alleviating inflammation symptoms than those based on Arnicae flos only.

## 2. Results

### 2.1. Sesquiterpene Lactones and Thymol Composition of Arnicae Planta Tota and Arnica Flower Extracts

**Figure 1:**
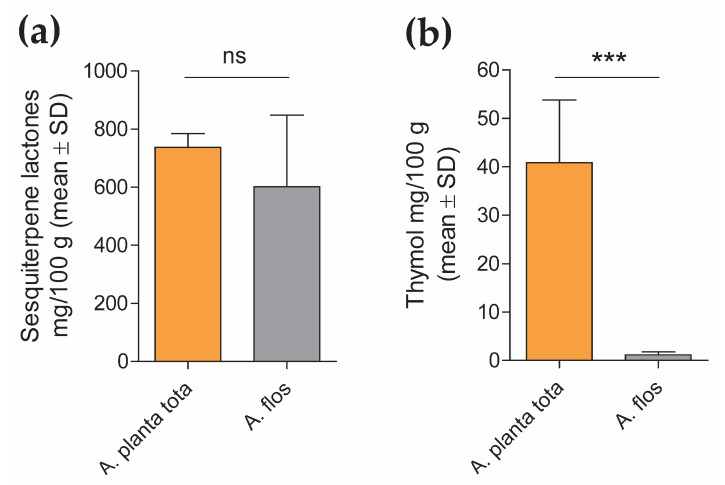
Sesquiterpene lactones (**a**) and thymol (**b**) content in Arnicae planta tota and Arnicae flos. Sesquiterpene lactones and thymol content was determined by HPLC from duplicate measurements of five independent extract preparations of Arnicae planta tota and Arnicae flos. Data (mean ± SD of five batch quantifications) are expressed as the amount of analyte (mg of sesquiterpene lactones or thymol) per 100 g Arnica extract. Differences in content of the measured compounds between Arnicae planta tota and Arnicae flos were compared using an unpaired *t*-test [*p* = 0.2612 in (**a**), *p* = 0.0001 (***) in (**b**)]. Abbreviation: A. planta tota, Arnicae planta tota; A. flos, Arnicae flos; ns, statistically non-significant.

The content of biologically active substances varies in different parts of the Arnica plant [1,6,7]. Sesquiterpene lactones and thymol are among the most abundant and bioactive substances of *Arnica montana* [1,2,3,5,6,7,8,10,15]. A preliminary analysis of Arnicae planta tota and Arnicae flos extracts using ultra-high-performance liquid chromatography with quadrupole time-of-flight tandem mass spectrometry (UHPLC-UV-hr-qTOF/MS) confirmed differences in the composition of both extracts (Figure S1a,b), including in the relative abundance of sesquiterpene lactones and thymol derivatives (Figure S1c). We further quantified the content of sesquiterpene lactones and thymol in Arnicae planta tota and Arnicae flos using high-performance liquid chromatography (HPLC) in five independent preparations of Arnicae planta tota and Arnicae flos extracts (Figure 1). While the content in sesquiterpene lactones (the sum of the detected helenalin and dihydrohelenalin derivatives) was not significantly different in both types of extracts (Figure 1a), thymol was significantly more abundant in Arnicae planta tota, compared to Arnicae flos (Figure 1b). An increased content of thymol in Arnicae planta tota vs. Arnicae flos was expected, given the known abundance of thymol in the roots of Arnica plant [1,7].

We next investigated whether differences in bioactive substance content might be associated with differences in the anti-inflammatory properties of both types of extracts. *A. montana* is well known for its ability to inhibit the activity of NF-κB and its downstream effectors 5-LO and COX-2 [1,2,8,9,16]. Hence, we compared the inhibitory effect of Arnicae planta tota and Arnicae flos on these NF-κB-mediated signaling pathways, using a variety of *in vitro*, cell-based and *in vivo* models.

### 2.2. Inhibition of NF-κB Activation by Arnicae Planta Tota and Arnica Flower Extracts

**Figure 2:**
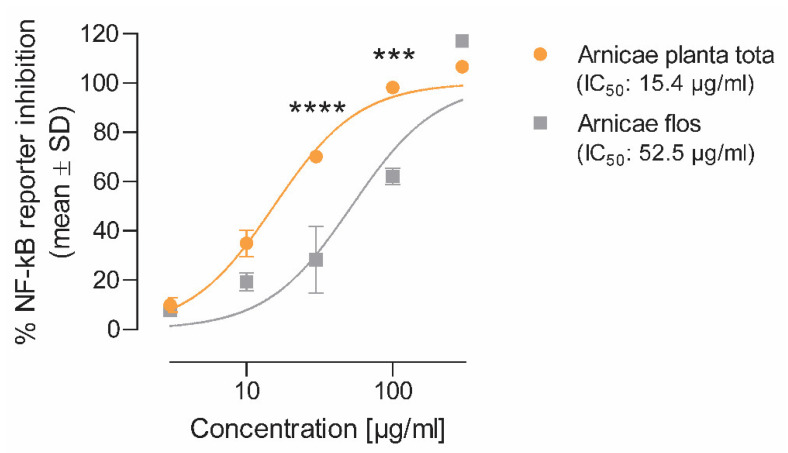
Inhibition of NF-κB activity by Arnicae planta tota and Arnicae flos. The inhibitory effect of increasing concentrations of Arnicae planta tota and Arnicae flos on NF-κB activity was evaluated using a NF-κB reporter assay in human T lymphocytic Jurkat cells. Data are expressed as the percentage of reporter inhibition relative to vehicle, and the respective half-maximal inhibitory concentrations (IC_50_) are indicated. Data are given as mean ± SD of two biological replicates from one representative experiment out of two independent experiments. Differences in NF-κB activity inhibition between Arnicae flos and Arnicae planta tota for each extract concentration were tested using a two-way ANOVA with Bonferroni’s correction for multiple comparisons (***, *p* < 0.001; ****, *p* < 0.0001). Positive control for NF-κB inhibition and cytotoxicity controls are shown in Figure S2.

The ability of Arnicae planta tota and Arnicae flos to inhibit NF-κB activation was evaluated in human T lymphocytic Jurkat cells stably transfected with a NF-κB reporter construct. Cells were preincubated with *A. montana* extracts or vehicle for 20 min and stimulated with calcium ionophore A23187 and phorbol 12-myristat 13-acetat (PMA) for 4 h. NF-κB activity was reported relative to that of the vehicle control. Both Arnicae planta tota and Arnicae flos inhibited NF-κB activation in a concentration-dependent manner, with a half-maximal inhibitory concentration (IC_50_) of 15.4 μg/mL for Arnicae planta tota vs. 52.5 μg/mL for Arnicae flos (Figure 2). The comparison of NF-κB inhibition by Arnicae planta tota and Arnicae flos showed that inhibition by Arnicae planta tota was significantly greater than that by Arnicae flos at 30 and 100 μg/mL extract (*p* < 0.0001 and *p* < 0.001, respectively), (Figure 2).

As positive control, cyclosporine A inhibited NF-κB activation with a IC_50_ of 6.79 nM (Figure S2a). We also verified that the inhibitory effect of *A. montana* extracts was not due to cytotoxicity (Figure S2b,c).

### 2.3. Inhibition of ALOX5 and PTGS2 Gene Expression by Arnicae Planta Tota and Arnicae Flos

**Figure 3:**
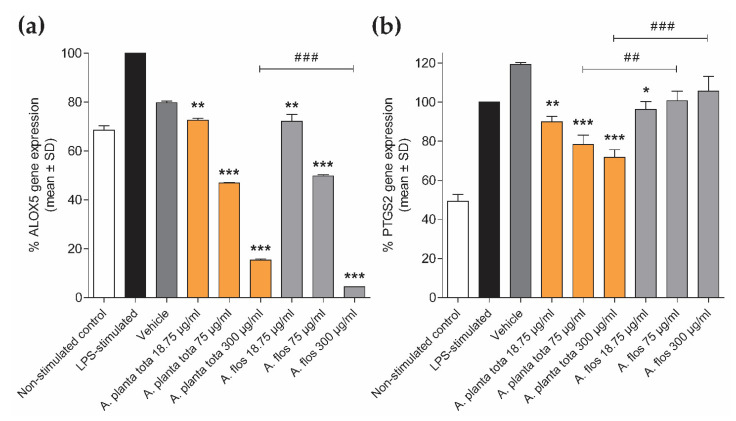
Inhibition of ALOX5 (**a**) and PTGS2 (**b**) gene expression by Arnicae planta tota and Arnicae flos in LPS-stimulated THP-1 cells. Human monocytic THP-1 cells were differentiated into macrophages, pre-treated for 4 h with increasing concentrations of Arnicae planta tota and Arnicae flos, and stimulated for 24 h with LPS before gene expression analysis using RT-qPCR. Data are expressed as the percentage of inhibition of ALOX5 and PTGS2 gene expression relative to the LPS-stimulated control. Data are given as mean ± SD of three biological replicates from one representative experiment out of two independent experiments. Differences in gene expression in Arnica-treated conditions vs. vehicle control were tested using a one-way ANOVA followed by Dunnett’s multiple comparison test (*, *p* < 0.05; **, *p* < 0.01; ***, *p* < 0.001). A further comparison of gene expression inhibition at the respective concentrations of Arnicae planta tota vs. Arnicae flos showed a significantly stronger inhibition of ALOX5 expression at 300 μg/mL Arnicae planta tota vs. Arnicae flos (# # #, *p* < 0.001), and a stronger inhibition of PTGS2 expression at 75 μg/mL Arnicae planta tota vs. Arnicae flos (# #, *p* < 0.01) and at 300 μg/mL Arnicae planta tota vs. Arnicae flos (# # #, *p* < 0.001). Abbreviations: ALOX5, arachidonate 5-lipoxygenase; A. planta tota, Arnicae planta tota; A. flos, Arnicae flos; LPS, lipopolysaccharide; PTGS2, prostaglandin-endoperoxide synthase 2. Cytotoxicity control is shown in Figure S3.

We next evaluated the effect of Arnicae planta tota and Arnicae flos on the expression of the NF-κB target genes ALOX5 and PTGS2 [17,18,27,28,29]. ALOX5 and PTGS2 encode the 5-LO and COX-2 enzymes, which initialize the conversion of arachidonic acid into leukotrienes and prostaglandins, respectively [16,18]. The expression of ALOX5 and PTGS2 was investigated in LPS-stimulated differentiated THP-1 macrophages, as an experimental system [29,30,31]. Human monocytic THP-1 cells were differentiated into macrophages with PMA for 72 h, pretreated with Arnicae planta tota and Arnicae flos (or vehicle) for 4 h and further stimulated with LPS for 24 h. Expression of ALOX5 and PTGS2 was measured using quantitative RT-PCR (Figure 3). Expression of both genes was inhibited by *A. montana* extracts in a concentration-dependent manner, albeit to a different extent. While expression of ALOX5 was comparably inhibited by Arnicae planta tota and Arnicae flos (Figure 3a), PTGS2 expression was preferably inhibited by Arnicae planta tota (Figure 3b). Moreover, while *A. montana* extracts inhibited both LPS-induced and basal expression of ALOX5 (Figure 3a), Arnicae planta tota only inhibited LPS-induced expression of PTGS2 (68% of inhibition above baseline relative to vehicle control; Figure 3b) in differentiated macrophages. Importantly, inhibition of gene expression by *A. montana* extracts was not due to cytotoxicity effects (Figure S3).

### 2.4. In Vitro Inhibition of 5-LO and COX-2 Enzymatic Activity by Arnicae Planta Tota and Arnicae Flos

**Figure 4:**
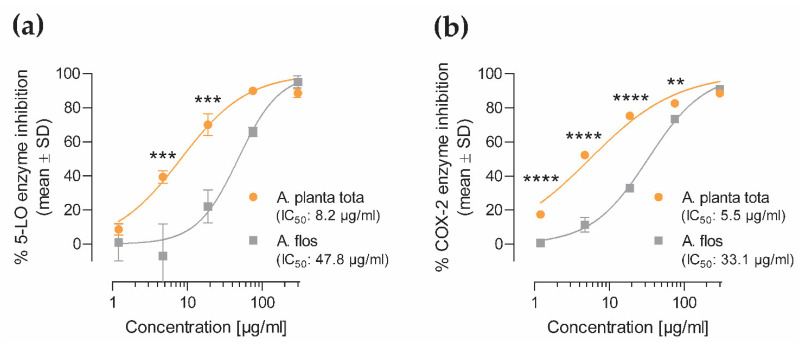
Inhibition of 5-LO (**a**) and COX-2 (**b**) *in vitro* enzymatic activity by Arnicae planta tota and Arnicae flos. The inhibitory effect of increasing concentrations of Arnicae planta tota and Arnicae flos on the enzymatic activity of human 5-lipoxygenase (5-LO) and cyclooxygenase-2 (COX-2) was tested using cell-free enzymatic assays. Data are expressed as the percentage of enzyme inhibition relative to vehicle. Data are given as mean ± SD of two biological replicates from one representative experiment out of two independent experiments. Differences in enzyme inhibition between Arnicae flos and Arnicae planta tota for each extract concentration was tested using a two-way ANOVA with Bonferroni’s correction for multiple comparisons (**, *p* < 0.01; ***, *p* < 0.001; ****, *p* < 0.0001). Abbreviations: A. planta tota, Arnicae planta tota; A. flos, Arnicae flos; 5-LO, 5-lipoxygenase; COX-2, cyclooxygenase-2. Positive controls for 5-LO and COX-2 inhibition are shown in Figure S4.

Enzymatic activity of 5-LO and COX-2 proteins, encoded by the ALOX5 and PTGS2 genes, has been shown to be directly inhibited by natural compounds, including by the sesquiterpene lactones of *A. montana* [16,18]. Hence, we investigated the potential inhibitory activity of Arnicae planta tota and Arnicae flos on the enzymatic activity of purified 5-LO and COX-2 proteins in cell-free enzymatic assays. Human recombinant 5-LO and COX-2 proteins were pre-incubated with *A. montana* extracts and their enzymatic activity was measured upon the addition of arachidonic acid as substrate (Figure 4). Both Arnicae planta tota and Arnicae flos inhibited 5-LO (Figure 4a) and COX-2 (Figure 4b) enzymatic activity in a concentration-dependent manner. The IC_50_ of Arnicae planta tota was lower than that of Arnicae flos in both cases (8.2 μg/mL vs. 47.8 μg/mL for 5-LO [Figure 4a] and 5.5 μg/mL vs. 33.1 μg/mL for COX-2 [Figure 4b], respectively). The comparison of enzymatic inhibition by Arnicae planta tota and Arnicae flos showed that inhibition by Arnicae planta tota was significantly greater than that by Arnicae flos at most tested concentrations (*p* < 0.01, *p* < 0.001 or *p* < 0.0001) (Figure 4).

Inhibition of *in vitro* enzymatic activity by well-characterized inhibitors (NDGA for 5-LO and rofecoxib for COX-2) used as positive controls, demonstrated expected IC_50_ values (Figure S4).

### 2.5. Inhibition of Arachidonic Acid Metabolite (Eicosanoid) Release from Human Primary Cells by Arnicae Planta Tota and ARNICAE flos

**Figure 5:**
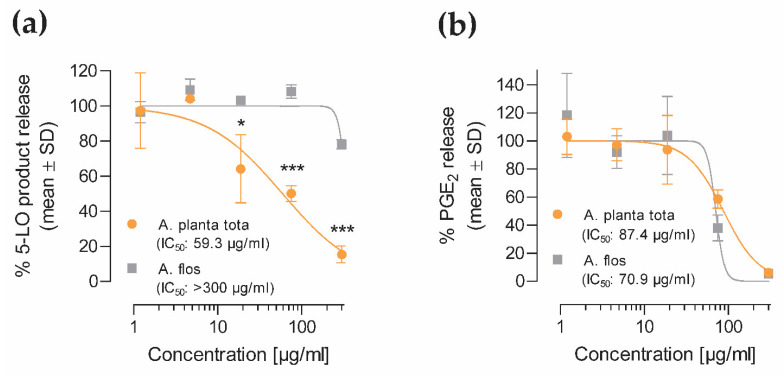
Inhibition of 5-LO (**a**) and COX-2 (**b**) product formation by Arnicae planta tota and Arnicae flos in isolated human peripheral blood cells. The inhibitory effect of Arnicae planta tota and Arnicae flos on 5-LO and COX-2 product formation was investigated in polymorphonuclear leukocytes (PMNL) (**a**) or in monocytes (**b**) isolated from peripheral blood of two (**a**) or three (**b**) healthy donors. Cells were pre-incubated with increasing concentrations of Arnicae planta tota and Arnicae flos for 15 min, then stimulated with either Ca^2+^-Ionophore A23187 in the presence of arachidonic acid for 10 min (**a**) or with LPS for 6 h (**b**). 5-LO product formation (sum of LTB_4_, its trans-isomers, and 5-HETE) was analysed using RP-HPLC (**a**) while COX-2 product formation (PGE_2_) was measured using UPLC-MS/MS (**b**). Data (mean ± SD of two or three donors, separately), are expressed as the percentage of released products relative to vehicle control. Presented data are from one representative experiment out of two independent experiments. Differences in product release inhibition between Arnicae flos and Arnicae planta tota for each extract concentration was tested using a two-way ANOVA with Bonferroni’s correction for multiple comparisons (*, *p* < 0.05; ***, *p* < 0.001). Abbreviations: A. planta tota, Arnicae planta tota; A. flos, Arnicae flos; 5-LO, 5-lipoxygenase; PGE_2_, prostaglandin E_2_. Positive controls for 5-LO and COX-2 product release, and cytotoxicity controls are shown in Figure S5.

Having detected an inhibitory effect of *A. montana* extracts on the expression of the genes encoding 5-LO and COX-2 (Figure 3) and on their enzymatic activity *in vitro* (Figure 4), we next assessed whether these inhibitory effects were reflected in their cellular enzymatic activity in human primary polymorphonuclear leukocytes (PMNL) and monocytes. These cells are key producers of pro-inflammatory arachidonic acid metabolites (eicosanoids) and as such are important mediators of inflammation *in vivo* [11,12,32,33]. PMNL and monocytes were isolated from the peripheral blood of healthy individuals. Freshly isolated cells were pre-incubated for 15 min with Arnicae planta tota and Arnicae flos extracts (or vehicle) and stimulated either with calcium ionophore A23187 in the presence of arachidonic acid for 10 min (PMNL) or with LPS for 6 h (monocytes). Arachidonic acid metabolites released in the cell culture supernatants were analysed and quantified by liquid chromatography methods. The 5-LO products leukotriene B_4_ (LTB_4_) and its trans-isomers, and 5-hydroxyeicosatetraenoic acid (5-HETE) were quantified in PMNL supernatants (Figure 5a), and the COX-2 product prostaglandin E_2_ (PGE_2_) was measured in monocyte supernatants (Figure 5b). Treatment of human primary PMNL with Arnicae planta tota resulted in a stronger inhibition of 5-LO product release than treatment with Arnicae flos, with IC_50_ of 59.3 μg/mL for Arnicae planta tota vs. >300 μg/mL for Arnicae flos (Figure 5a). Inhibition by Arnicae planta tota was significantly greater than that by Arnicae flos for extract concentrations ≥18.8 μg/mL (*p* < 0.05 to *p* < 0.001) (Figure 5a). On the other hand, Arnicae planta tota and Arnicae flos inhibited PGE_2_ release from LPS-stimulated monocytes in a comparable manner, with IC_50_ of 87.4 μg/mL and 70.9 μg/mL, respectively (Figure 5b). The reference inhibitors (zileuton for 5-LO and indomethacin for COX-2) tested in parallel, demonstrated, as expected, a strong inhibition of arachidonic acid metabolite release (Figure S5a,b). The inhibition mediated by *A. montana* extracts in that concentration range (up to 75 μg/mL) was not due to cytotoxicity (Figure S5c,d). It should be noted that partial toxicity was observed in monocytes at a concentration of 300 μg/mL of either Arnicae planta tota or Arnicae flos (Figure S5c).

### 2.6. Inhibition of Inflammation Symptoms by Arnicae Planta Tota and Arnicae Flos in a Mouse Model of Carrageenan-Induced Paw Oedema

**Figure 6:**
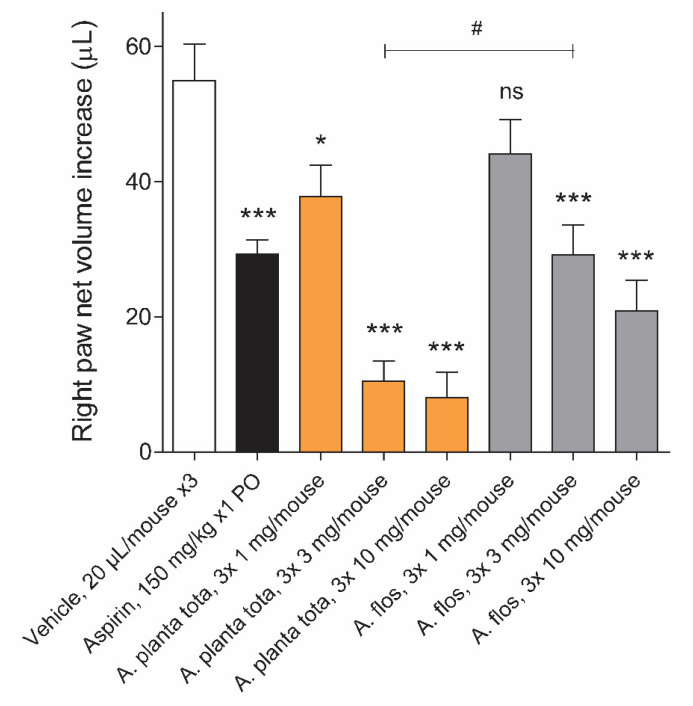
Inhibition of carrageenan-induced paw oedema in mice treated with Arnicae planta tota or Arnicae flos (*n* = 6). The indicated amounts of Arnicae planta tota and Arnicae flos were applied topically to the right hind paw of male ICR mice three times at 20 min intervals before intraplantar carrageenan injection. The positive control aspirin was given orally (PO) 1 h before carrageenan injection. Right hind paw oedema was recorded 4 h after carrageenan injection. Uninjected left hind paw served as internal negative control. Data are expressed as right-hind-paw net volume increase (μL) and represent mean ± SD paw volume increase of six mice treated in parallel (one representative experiment out of two independent experiments). Differences in paw swelling in treated conditions vs. vehicle control were tested using a one-way ANOVA followed by Dunnett’s multiple comparison test (*, *p* < 0.05; ***, *p* < 0.001). A further comparison of swelling at the respective concentrations of Arnicae planta tota vs. Arnicae flos showed a significantly reduced swelling at 3 × 3 mg/mouse Arnicae planta tota vs. Arnicae flos (#, *p* < 0.05). Abbreviations: A. planta tota, Arnicae planta tota; A. flos, Arnicae flos; ns, statistically non-significant.

Carrageenan is a well-acknowledged pro-inflammatory agent used to initiate an acute inflammatory response upon injection into the footpad of rodents [34,35,36]. This carrageenan-induced paw oedema animal model is the experimental system of choice for evaluating the anti-inflammatory effect of drugs or natural products such as *A. montana* extracts. We investigated the anti-inflammatory properties of Arnicae planta tota and Arnicae flos upon three consecutive topical applications (of 1 mg, 3 mg, or 10 mg per mouse, each) to the right hind paw of ICR mice, prior to the intraplantar injection of carrageenan. Vehicle served as a negative control for oedema inhibition. Aspirin administered orally (150 mg/kg) prior to the carrageenan injection served as a positive control (reference inhibitor). A total of forty-eight mice divided into eight treatment groups (six mice per group) were included in this analysis. Foot swelling was measured 4 h after the carrageenan injection (Figure 6). Topical application of both Arnicae planta tota and Arnicae flos extracts resulted in a dose-dependent reduction in paw swelling, compared to the vehicle control. Maximum inhibition of paw oedema was observed with three applications of 10 mg *A. montana* extract, with an 85% and 62% reduction in foot swelling relative to the vehicle for Arnicae planta tota and Arnicae flos, respectively. In comparison, a single oral dose of aspirin (150 mg/kg) reduced foot swelling by 47%, compared to the vehicle (Figure 6). The reduction of the paw oedema by Arnicae planta tota was statistically significant (vs. vehicle), even at the lower applied dose of 3 x 1 mg/mouse (*p* < 0.05), while the difference from the vehicle for higher doses of *A. montana* extracts was significant for both Arnicae planta tota and Arnicae flos (*p* < 0.001) (Figure 6). A comparison of paw swelling at the respective doses of Arnicae planta tota vs. Arnicae flos showed a significantly reduced swelling at 3 x 3 mg Arnicae planta tota vs. Arnicae flos per mouse (#, *p* < 0.05) (Figure 6).

## 3. Discussion

While the anti-inflammatory activity of Arnicae flos is well described in the literature [1,2,8,9,16], data on Arnicae planta tota are scarce [25,26]. This study compared the anti-inflammatory properties of Arnicae planta tota to those of Arnicae flos, using a variety of *in vitro* and *in vivo* assays.

**Figure 7:**
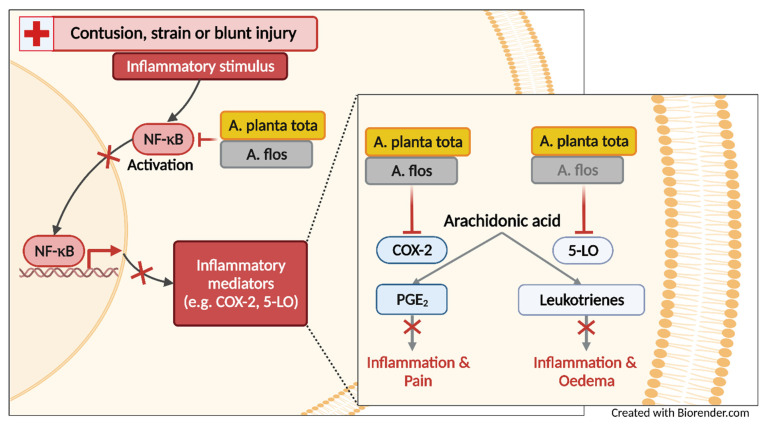
Inhibition of the NF-κB—eicosanoid inflammation pathway by *Arnica montana* extracts, as reported in this study. Arnicae planta tota and Arnicae flos both inhibited NF-κB activation, the expression and activity of 5-LO and COX-2 enzymes, and the release of eicosanoids (leukotrienes and prostaglandin PGE_2_), to varying extents. Topical application of Arnicae planta tota and Arnicae flos also resulted in reduced paw oedema induced by carrageenan. Overall, the anti-inflammatory activity of Arnicae planta tota was either comparable to or stronger than that of Arnicae flos. Abbreviations: A. planta tota, Arnicae planta tota; A. flos, Arnicae flos; 5-LO, 5-lipoxygenase; COX-2, cyclooxygenase-2; NF-κB, nuclear factor kappa B; PGE_2_, prostaglandin E_2_. Figure was created with BioRender.com (accessed on 13 December 2022).

We showed that in most models of inflammation investigated, both Arnicae planta tota and Arnicae flos exerted inhibitory functions toward tested pro-inflammatory targets, and that Arnicae planta tota was a stronger inhibitor compared to Arnicae flos (lower IC_50_). Arnicae planta tota inhibited NF-κB activation in a reporter assay and the LPS-induced expression of the NF-κB target genes ALOX5 and PTGS2 in differentiated THP-1 cells (Figure 7). The enzymatic activity of the proteins encoded by ALOX5 and PTGS2 (5-LO and COX-2, respectively,) was also directly inhibited by Arnicae planta tota in a cell-free assay, demonstrating a second level of inhibition downstream of NF-κB activation (Figure 7). Inhibition of 5-LO and COX-2 enzymatic activity was confirmed in human primary cells, as evidenced by the reduced release of the proinflammatory arachidonic acid metabolites leukotrienes and prostaglandins, in cells pretreated with Arnicae planta tota (Figure 7). Finally, topical application of Arnicae planta tota (and Arnicae flos) partly prevented foot swelling in a mouse model of carrageenan-induced paw oedema (Figure 7). The anti-oedema activity of Arnica is in line with its inhibitory effect on the production of leukotrienes and prostaglandins, which are both directly involved in oedema formation [16,18,19,20,23]. Our results therefore confirm the anti-inflammatory activities attributed to Arnicae flos [1,2,8,9], while demonstrating—to the best of our knowledge, for the first time—superior anti-inflammatory activities for Arnicae planta tota due to interference with the NF-κB—eicosanoid inflammation pathway. The superior anti-inflammatory properties of Arnicae planta tota are likely due to the increased and broader content of biologically active substances in the whole Arnica plant, compared to the flowers alone [1,2,3,4,5,6,7,8,9]. The stronger inhibitory effect of Arnicae planta tota on leukotriene release in PMNL, and its stronger anti-oedema effect in the carrageenan-induced inflammation mouse model, suggest that leukotriene-mediated inflammation might be a major target of the anti-inflammatory activity of Arnicae planta tota, compared to Arnicae flos.

In addition to anti-inflammatory activities, we also unveiled an unexpected inhibitory effect of Arnicae planta tota on the basal expression of ALOX5 in differentiated THP-1 cells. This effect was also observed upon treatment with Arnicae flos. The transcription factors responsible for basal ALOX5 expression in differentiated THP-1 cells that are targeted for inhibition by *A. montana* extracts are yet to be identified. Previous studies indicated that tandem consensus-binding motifs for the Egr-1/Sp1 transcription factors within ALOX5 core promoter are essential for its basal activity [29,37,38]. Additional studies would be necessary to determine whether Egr-1/Sp1 transcription factors are inhibited by *A. montana* extracts.

Interestingly, while LPS-induced expression of PTGS2 in differentiated THP-1 was inhibited by Arnicae planta tota in a concentration-dependent manner, it remained unaffected by pre-treatment with Arnicae flos. This suggests that some bioactive substances contained in Arnicae planta tota but not in Arnicae flos were responsible for this inhibition. Indeed, bioactive substance content varies in different parts of the plant [1,6,7], as confirmed by our preliminary HPLC experiments. Whether it is thymol, which was detected in a higher amount in Arnicae planta tota compared to Arnicae flos, and is known for its anti-inflammatory, antioxidant, and anti-microbial properties [1,7,10,11,12,13,14,15], or other substances within Arnicae planta tota which are responsible for the inhibition of LPS-induced PTGS2 in differentiated THP-1 cells, remains to be uncovered.

It is worth noting that, although ALOX5 and PTGS2 have been described as NF-κB targets [16,18], we have not demonstrated that LPS-induced expression of these genes in differentiated THP-1 cells is actually mediated by NF-κB. In fact, a recent study showed that LPS-induced expression of ALOX5 in differentiated THP-1 cells does not involve NF-κB but an as-yet-uncharacterized factor [29]. If LPS-induced expression of PTGS2 in these cells is also independent of NF-κB, it would explain the absence of inhibition of LPS-induced expression of PTGS2 by Arnicae flos. Whatever the mechanism of induction of these genes in response to LPS is, our results emphasize the difference in the inhibitory effect of Arnicae planta tota and Arnicae flos.

A strength of our study is the use of different *in vitro* and *in vivo* experimental systems directly comparing planta tota and flos extracts prepared from the same source of *A. montana* and employing the same extraction procedure. The data obtained in the mouse model of acute inflammation using a mode of application comparable to that typically used in humans (topical application) are very appealing, and should now be reproduced in humans. It is worth noting that the dose of extract applied locally in the mouse model was in a range comparable to that recommended for existing Arnica ointments (e.g., Arnicae planta tota 30% ointment, available in Germany). Considering a recommended average application of 2 mg/cm^2^, three to five times a day, an amount of 1.8 to 3 mg Arnicae planta tota would be applied locally, using Arnica 30% ointment (compared to the 3 to 30 mg Arnicae planta tota applied in the mouse model of acute inflammation, described in this study). In addition to validating its use in preventing local inflammation (i.e., following a pre-treatment protocol, as described in this study), it would also be interesting to evaluate the potential benefit of the topical application of Arnicae planta tota to treat local inflammation (i.e., following induced inflammation), as shown for other natural products in the paw oedema mouse model [36].

## 4. Materials and Methods

### 4.1. Arnica Montana Planta Tota and Flos Extracts

Preparation of dry extracts from *Arnica montana* L., planta tota and *Arnica montana* L., flos, organically cultivated in Germany on fields dedicated to medicinal-plant cultivation, was conducted using liquid extracts from Arnicae planta tota fresh plant or Arnicae flos fresh flowers (Arnicae planta tota recens, ethanolic extract 1:1.1, ethanol 30% [m/m] and Arnicae flos recens, ethanolic extract 1:1.1, ethanol 30% [m/m]). The dry herbal extracts were dissolved using 50% ethanol/50% purified water (m/m), resulting in a 30 mg/mL stock solution. The suspension was vortexed for 10 min and sonicated for 30 min with occasional vortexing. Samples were centrifuged for 10 min at 3000 x g. The supernatant was carefully harvested and used for experiments. If not stated otherwise, 50% ethanol/50% purified water (m/m), diluted to a final concentration of 0.5% ethanol, served as vehicle control in the respective assays.

### 4.2. Characterisation of A. Montana Extracts by HPLC

#### 4.2.1. Composition and Semi-Quantitative Analysis of A. Montana Extracts

The composition of Arnicae planta tota and Arnicae flos extracts was evaluated using ultra-high-performance liquid chromatography with quadrupole time-of-flight tandem mass spectrometry (UHPLC-UV-hr-qTOF/MS). Each sample was measured in the form of technical triplicates. A total of 100 mg of each dry extract was dissolved in 10 mL, 1:1, v:v, acetonitrile:water containing 1% formic acid, vortexed for 2 min and ultrasonicated for 15 min. Finally, 1:50 v:v dilution was performed for each extract in 1:1, v:v, acetonitrile:water containing 1% formic acid, and subjected to instrumental analysis.

Chromatographic separation was performed on a Thermo Scientific Dionex UltiMate 3000 (ThermoFisher Scientific, Waltham, MA, USA), using an RRHD Zorbax C18 (2.1 × 100 mm, 1.8 μm) column (Agilent Technologies, Santa Clara, CA, USA). The mobile phase consisted of acetonitrile (B) and 0.1% formic acid in water (A). The flow rate was 0.4 mL/min, and the injection volume was 5 μL. The column oven was set at 45 °C. The following gradient was used: 0.0 min 3% B, 15.0 min 50% B, 18.0 min 80% B, 19 min 100% B, 21.0 min 100% B, 21.5 min 3 % B, and 23 min 3 % B.

The eluate from the liquid chromatography was directly introduced into the maXis Impact Ultra High Resolution TOF-MS mass spectrometer (Bruker Daltonics, Bremen, Germany), with mass scanning from 50–800 m/z and spectra rate 4 Hz, using electrospray ionization (ESI) in positive mode. The mass accuracy before each run was verified by comparison with sodium formate adducts. The mass accuracies were rounded to 1 mDa, and the corresponding retention times to 0.05 min. The UV spectra were recorded at 280 nm. Interpretation of the mass signals was conducted using Compass Data Analysis 4.2 (Bruker, Billerica, MA, USA). Identification of the detected sesquiterpene lactones and thymol derivatives was performed, based on the reported literature [39,40,41], and their characteristic transition and relative abundance was determined according to the respective peak area.

#### 4.2.2. Quantification of Sesquiterpene Lactones and Thymol Content in A. Montana Extracts

The content of sesquiterpene lactones and thymol in Arnicae planta tota and Arnicae flos extracts was quantified by high-performance liquid chromatography (HPLC), using five independent batches of extract preparation for each. Each HPLC measurement was carried out in duplicate.

For sesquiterpene lactones quantification, a modified HPLC method adapted from the monograph “Arnica tincture” of the European Pharmacopoeia (Ph. Eur.) 10.0/1809 was used, as follows. Arnica dry extracts (0.1 g) were dissolved in 5 mL ethanol R2 30% (*w*/*w*) and mixed with 1 mL internal standard solution (1 mg/mL santonin (chemical reference substance, European Directorate for the Quality of Medicines & HealthCare (EDQM), Council of Europe, Strasbourg, France), 2 mg/mL butyl 4-hydroxybenzoate R in methanol R2) and 3 g anhydrous aluminum oxide R. The mixture was shaken for 120 s, filtered through filter paper and evaporated to dryness under reduced pressure in a water bath. The desiccated residue was dissolved in 2 mL 50% methanol R2/50% chromatography-grade water R, and filtered through a 0.45 μm PVDF membrane filter. HPLC analysis was performed on an Ultimate 3000 RSLC 02 HPLC system equipped with a UV detector (ThermoFisher Scientific, Waltham, MA, USA), using a Symmetry C18 Column (150 mm length × 3.9 mm inner diameter, 5 μm particle size; Waters). The injection volume was 20 μL, the flow rate was 1.0 mL/min, the column temperature was maintained at 22 °C, and the detection wavelength of quantification was set at 225 nm. The mobile phase for chromatography was distilled water R (A) and methanol R2 (B). Following column equilibration in 40% methanol for 5 min, separation was obtained by gradient elution through increasing the methanol from 40% to 45% linearly over 17 min and holding for 10 min, followed by a second gradient, from 45% to 55% methanol for 25 min. The column was then washed by increasing the methanol to 90% for 1 min, and held for another 1 min, before re-equilibration at starting mobile-phase conditions (40% methanol in chromatography-grade water R). Retention time was about 10 min for santonin and about 49 min for butyl 4-hydroxybenzoate. Data were interpreted based on peak area determination (the sum of the peaks of helenalin and dihydrohelenalin derivatives between the reference substances santonin and butyl 4-hydroxybenzoate) and the santonin internal standard, using the Chromeleon 7.2 Chromatography Data System software (ThermoFisher Scientific, Waltham, MA, USA). Sesquiterpene lactone content was calculated according to the santonin and dihydrohelenalin tiglate peak area, using the conversion factor 1.187, as recommended by the Ph. Eur. 10.0/1809. Data (mean ± SD of five analyte batch quantification, each expressed as mean of duplicate measurement) were expressed in mg sesquiterpene lactones/100 g Arnica extract.

For thymol quantification, Arnica dry extracts (0.05 g) were dissolved in 50% methanol R2, mixed with 0.8 mL internal standard solution (1 mg/mL carvacrol [primary reference standard, Sigma-Aldrich/Merck, 04270590, Darmstadt, Germany] in methanol R2), and diluted to 100 mL with 50% methanol R2. The mixture was filtered through a 0.45 μm PVDF membrane. A solution containing 0.2 μg/mL thymol (primary reference standard, PhytoLab, 89287, Vestenbergsgreuth, Germany) in 50% methanol R2 was also measured as the reference standard. HPLC analysis was performed on an Ultimate 3000 RSLC 02 HPLC system equipped with an RF-20A fluorescence detector (ThermoFisher Scientific, Waltham, MA, USA), using a SunFire C18 Column (150 mm length × 3.0 mm inner diameter, 3.5 μm particle size; Waters). The injection volume was 10 μL, the flow rate was 1.0 mL/min, the column temperature was maintained at 30 °C, and detection was performed at 280 nm/310 nm. The mobile phase for chromatography was 0.1% trifluoroacetic acid R (A) and chromatography-grade acetonitrile R2 (B). Following equilibration in 40% acetonitrile (B) / 60% 0.1% trifluoroacetic acid (A) for 2 min, separation was obtained by gradient elution through increasing the acetonitrile from 40% to 65% linearly for 8 min. The column was then washed by increasing the acetonitrile to 95% for 2 min, and held for another 3 min, before re-equilibration at starting mobile-phase conditions. Retention time was about 9.0 min for carvacrol and about 9.6 min for thymol. Data were interpreted based on peak height using internal standard correction, with the Chromeleon 7.2 Chromatography Data System software (ThermoFisher Scientific, Waltham, MA, USA). Thymol content in the Arnica extracts was calculated according to the thymol external reference standard. Data (mean ± SD of the quantification of the five analyte batches) were expressed in mg thymol/100g Arnica extract.

### 4.3. NF-κB Reporter Assay

The inhibitory effect of Arnicae planta tota and Arnicae flos on NF-κB activity was measured using an NF-κB reporter assay (Eurofins Panlabs #361000-1, New Taipei City, Taiwan). Briefly, human T lymphocytic Jurkat cells (2 × 10^6^ cells/mL) stably transfected with a construct comprising a NF-κB response element controlling the transcription of the beta-galactosidase reporter gene (Eurofins Panlabs Taiwan #C13900, New Taipei City, Taiwan) were preincubated for 20 min at increasing concentrations (3–300 μg/mL in 0.5% ethanol final concentration) of either Arnicae planta tota or Arnicae flos extract, or vehicle (0.5% ethanol) in RPMI-1640 buffer pH 7.4 at 37 °C (two wells per condition). Increasing concentrations (3–300 nM) of cyclosporin A (Sigma-Aldrich/Merck C3662, Darmstadt, Germany), serving as positive control for NF-κB inhibition, were tested in parallel. Cells were then stimulated for 4 h with 0.5 μM Calcium Ionophore A23187 and 50 ng/mL phorbol 12-myristat 13-acetat (PMA), and harvested to quantify reporter-gene activity. Beta-galactosidase reporter-gene activity was determined by the conversion of fluorescein di-β-D-galactopyranoside (FDG; ThermoFisher Scientific, F1179, Waltham, MA, USA) to fluorescein, and the fluorescence intensity was measured on a SpectroFluor Plus plate reader. A decrease of ≥50% in fluorescence intensity, relative to 10 μM cyclosporin A, indicates significant inhibitory activity. Data (mean ± SD of duplicate cell treatment) were expressed as the percentage of reporter inhibition relative to vehicle, and the half-maximal inhibitory concentration (IC_50_) for Arnicae planta tota and Arnicae flos was calculated.

Cytotoxicity assays were conducted in triplicate under the same experimental conditions, as a control for specific NF-κB inhibition. Staurosporine (Sigma-Aldrich/Merck S4400, Darmstadt, Germany; 0.001–100 μg/mL) was tested in parallel (in duplicate) as positive control for cytotoxicity. Following cell stimulation, CellTiter-Glo reagent (CellTiter-Glo^®^ Luminescent Cell Viability Assay, Promega, Madison, WI, USA) was added to each well, and luminescence was measured on a Safire2 Microplate Reader (Tecan, Männedorf, Switzerland). CellTiter-Glo reagent measures the amount of ATP present in the cell-culture well. ATP is an indicator of metabolically active cells, and its amount is directly proportional to the number of living cells. The percentage of cell viability was calculated relative to the vehicle control.

### 4.4. ALOX5 and PTGS2 Gene Expression Analysis Using Quantitative RT-PCR

#### 4.4.1. THP-1 Cell Differentiation and Stimulation

Human monocytic leukemia THP-1 cells (American Type Culture Collection (ATCC) #TIB-202, Manassas, VA, USA) were seeded in a 48-well plate and incubated in culture medium (RPMI 1640 supplemented with 10% heat-inactivated fetal calf serum, 1% L-glutamine, and 1% penicillin-streptomycin) for 4 h. Cells were differentiated into macrophages by the addition of 200 nM PMA in culture medium for 72 h. PMA-containing medium was replaced by fresh medium, and differentiated cells were pretreated for 4 h with the indicated concentrations of Arnicae planta tota or Arnicae flos, or vehicle (0.5% ethanol) as control. Cells (three wells per condition) were further stimulated for 24 h with 100 ng/mL lipopolysaccharide (LPS).

#### 4.4.2. Cytotoxicity Control Assay

Cytotoxicity assays were conducted in triplicate under the same experimental conditions. Following stimulation, cells were incubated with WST-8 (CCK-8 #311KTA1020, Tebubio, Le Perray, France) and optical density was measured at 450 nm using a VERSAmax spectrophotometer (Molecular Devices, San Jose, CA, USA). WST-8 is a tetrazolium salt that is reduced in a yellow-colored formazan dye by mitochondrial succinate dehydrogenase (an indicator of metabolically active cells). Formazan product formation is directly proportional to the number of living cells and their metabolic activity. The percentage of cell viability was calculated relative to the LPS-stimulated condition.

#### 4.4.3. RNA Isolation and Quantitative RT-PCR

Following LPS stimulation, cells were harvested and stored at −80 °C until RNA isolation. Total RNA was isolated using TriPure Isolation Reagent^®^ (Sigma-Aldrich/Merck, Darmstadt, Germany) according to the supplier’s instructions. RNA quality was verified using capillary electrophoresis (Bioanalyzer 2100, Agilent Technologies, Santa Clara, CA, USA) and RNA concentration was measured using spectrophotometry (Synergy H1, BioTek Instruments/Agilent Technologies, Santa Clara, CA, USA). Complementary DNA (cDNA) was synthetized by reverse transcription of total RNA in the presence of oligo(dT) using Transcriptor Reverse Transcriptase (Roche, Basel, Switzerland). Quantitative PCR was performed using ONEGreen^®^ FAST qPCR Premix (Ozyme, Saint-Cyr-l’Ecole, France) according to the manufacturer’s instructions, and triplicate reactions were run on a LightCycler^®^ 480 System (Roche Molecular System Inc., Pleasanton, CA, USA), using a two-step protocol (95 °C 5 s, 60 °C 34 s; 45 cycles). The forward and reverse primers used for amplification of ALOX5 (NM_000698), PTGS2 (NM_000963) and the normalizer gene GAPDH (NM_002046) were designed using Primer3 and NCBI Blast, and were ordered from Sigma-Aldrich/Merck (Darmstadt, Germany). The respective sequences (5′–3′) of the forward and reverse primers were as follows: ALOX5, TCATCGTGGACTTTGAGCTG and CAGAAGGTGGGTGATGGTCT; PTGS2, TGAGCATCTACGGTTTGCTG and TGCTTGTCTGGAACAACTGC; GAPDH, GGCTCTCCAGAACATCATCCCTGC and GGGTGTCGCTGTTGAAGTCAGAGG. Cycle threshold (Ct) values were expressed in arbitrary units (AU) according to the formula: AU = (1/2^Ct^) × 10^6^. AU of the genes of interest ALOX5 and PTGS2 (AU_GOI_) were normalized to that of the housekeeping gene GAPDH (AU_GAPDH_), according to the formula AU_GOI_/AU_GAPDH_, and ALOX5 and PTGS2 expression data were expressed as a percentage of gene expression relative to that in the LPS-stimulated control.

### 4.5. 5-LO and COX-2 In Vitro Enzymatic Assays

The inhibitory effect of Arnicae planta tota and Arnicae flos on the enzymatic activity of human 5-lipoxygenase (5-LO) and cyclooxygenase-2 (COX-2) was assessed using cell-free enzymatic assays (Eurofins Cerep #772, Celle-Lévescault, France, and Eurofins Panlabs #118030, New Taipei City, Taiwan, respectively).

For the 5-LO enzymatic assay, 2.4 U/mL purified human recombinant 5-LO protein was preincubated in duplicate with increasing concentrations (1.2–300 μg/mL) of either Arnicae planta tota or Arnicae flos extract, or vehicle (0.5% ethanol), for 15 min at 25 °C in Tris buffer pH 7.4. Nordihydroguaiaretic acid (NDGA; Cayman Chemical 70,300-1, Ann Arbor, MI, USA; 0.01–30 µM) served as control inhibitor. The 5-LO enzymatic reaction was performed in the presence of 25 µM arachidonic acid (Biomol Cay90010-100, Hamburg, Germany), as substrate, and non-fluorescent dihydrorhodamine 123 for 5 min at 25 °C. The 5-LO enzymatic activity was measured using fluorescence spectroscopy at 500 nm/590 nm of rhodamine 123, generated by 5-LO-mediated conversion of dihydrorhodamine 123.

For the COX-2 enzymatic assay, 34 U/mL purified human recombinant COX-2 protein was preincubated in duplicate with increasing concentrations (1.2–300 μg/mL) of either Arnicae planta tota or Arnicae flos extract, or vehicle (0.5% ethanol), for 15 min at 25 °C in modified Tris-HCl buffer pH 8.0. Rofecoxib (Glentham Life Sciences GP0159, Corsham, UK; 0.03–3 µM) served as control inhibitor. The COX-2 enzymatic reaction was initiated by adding 3 µM arachidonic acid substrate and 100 µM Amplifu Red (10-Acetyl-3,7-dihydroxyphenoxazine), and further incubated for 3 min at 25 °C. The COX-2 enzymatic activity was analysed using fluorescence spectroscopy at 535 nm/590 nm of resorufin, produced by COX-2-catalysed oxidation of Amplifu Red.

Measurements were performed in duplicate, and data were expressed as the percentage of enzyme inhibition relative to vehicle. IC_50_ values were calculated for Arnicae planta tota and Arnicae flos, as well as for the reference inhibitors nordihydroguaiaretic acid (5-LO assay) and rofecoxib (COX-2 assay).

### 4.6. 5-LO and COX-2 Product Release from Human Peripheral Blood Cells

The inhibitory effect of Arnicae planta tota and Arnicae flos on 5-LO and COX-2 product formation was investigated in cellular assays.

#### 4.6.1. 5-LO Product Formation

For 5-LO product formation analysis, polymorphonuclear leukocytes (PMNL) were freshly isolated from human peripheral blood of two consenting healthy donors (Institute of Transfusion Medicine, University Hospital Jena, Germany) who had declared that they had not taken any anti-inflammatory drugs within 10 days prior to blood withdrawal, as previously described [42]. The experimental protocol was approved by the ethical committee of the Jena University Hospital, and all methods were performed in accordance with the relevant guidelines and regulations. PMNL (5 × 10^6^/^mL^) were preincubated with increasing concentrations (1.2–300 μg/mL) of either Arnicae planta tota or Arnicae flos extract, or vehicle (0.5% ethanol), for 15 min at 37 °C, and stimulated with 2.5 µM Ca^2+^-Ionophore A23187 (Biomol Cay11016-10, Hamburg, Germany) in the presence of 20 µM arachidonic acid (Biomol Cay90010-100, Hamburg, Germany), as 5-LO substrate, for 10 min at 37 °C. The 5-LO- specific inhibitor zileuton (Biosynth Ltd. FZ28760, Staad, Switzerland; 15 µM) was used as a reference compound. The 5-LO products LTB_4_, its trans-isomers, and 5-HETE released in the supernatant were analysed using reverse-phase high-performance liquid chromatography (RP-HPLC), as reported [42], their sum defining 5-LO products. Data (mean ± SD of two donors) were expressed as the percentage of released 5-LO products relative to vehicle control.

#### 4.6.2. COX-2 Product Formation (PGE_2_)

For COX-2 product formation analysis, monocytes were freshly isolated from human peripheral blood of three consenting healthy donors (Institute of Transfusion Medicine, University Hospital Jena, Germany) who had declared that they had not taken any anti-inflammatory drugs within 10 days prior to blood withdrawal, as previously described [42]. The experimental protocol was approved by the ethical committee of the Jena University Hospital and all methods were performed in accordance with the relevant guidelines and regulations. Cells (1 × 10^6^/^mL^) were preincubated with increasing concentrations (1.2–300 μg/mL) of either Arnicae planta tota or Arnicae flos extract, or vehicle (0.5% ethanol), for 15 min at 37 °C, and then stimulated with 100 ng/mL LPS for 6 h. Indomethacin (Sigma-Aldrich/Merck I7378, Darmstadt, Germany; 10 µM) was used as reference inhibitor. Cell culture supernatants of the three donors were collected, and the released COX-2 product PGE_2_ was analysed using ultra-performance liquid chromatography tandem mass spectrometry (UPLC-MS/MS), as previously reported [43]. Data (mean ± SD of three donors) were expressed as the percentage of released PGE_2_, relative to vehicle control.

To verify that the effect of Arnica extracts was not due to cytotoxicity during the 6-h incubation time, mitochondrial dehydrogenase activity was measured under comparable experimental conditions using an MTT-based assay. Briefly, monocytes were preincubated with increasing concentrations (1.2–300 μg/mL) of either Arnicae planta tota or Arnicae flos extract, 0.5% ethanol (vehicle), or 10 µM indomethacin (inhibition control) for 15 min at 37 °C, and stimulated with 100 ng/mL LPS for 6 h, as above. Following stimulation, 3-[4,5-dimethylthiazol-2-yl]-2,5-diphenyltetrazolium bromide (MTT; Sigma-Aldrich/Merck, Darmstadt, Germany) was added to each well for 2 h. An additional LPS-stimulated well was treated with 0.1% Triton X-100, as a cytotoxicity control (100% dead cells), before the addition of 2 mmol/L MTT. Cells were lysed, purple-colored formazan crystals were solubilized with 10% SDS in 20 mmol/L HCl, and absorbance was measured at 570 nm, using a Multiskan Spectrum Microplate Reader (ThermoFisher Scientific, Waltham, MA, USA). Data (mean ± SD of three donors) were expressed as the percentage of cell viability relative to the stimulated vehicle control.

### 4.7. Carrageenan-Induced Inflammation Mouse Model

Male ICR mice (26 ± 2 g, 6 mice per group), purchased from BioLasco Taiwan (Taipei, Taiwan; under Charles River Laboratories’ License) were fasted overnight before the start of treatment and for the duration of the experiment. Arnicae planta tota, Arnicae flos, given at 1, 3 and 10 mg/paw, or vehicle (50% ethanol), each in a volume of 20 µL, was applied topically to the right hind paw around the site of the carrageenan injection three consecutive times at 20 min intervals, starting 1 h before the carrageenan injection. The positive control Aspirin was given orally at a dosage of 150 mg/kg 1 h before the carrageenan injection. The mice received an intraplantar injection of carrageenan (50 µL of 1% suspension) into the right hind paw. Paw oedema was recorded 4 h after the carrageenan challenge, using a plethysmometer. The uninjected left hind paw served as an internal negative control. The animals were prohibited from licking their paws by wearing neck ruffs after drug application and for the duration of the experiment. The study was performed by Pharmacology Discovery Services (New Taipei City, Taiwan) under the Animal Care and Use Protocol No. IN018-10222020 approved by the Institutional Animal Care and Use Committee (IACUC) on October 10, 2020, in a laboratory animal facility accredited by the Association for Assessment and Accreditation of Laboratory Animal Care International (AAALAC), and in general accordance with the *Guide for the Care and Use of Laboratory Animals: Eighth Edition* (National Academies Press, Washington, D.C., 2011).

### 4.8. Statistical Analysis

Statistical analysis, curve fitting and IC_50_ value calculation were performed using GraphPad Prism version 9.2.0 (GraphPad Software Inc., San Diego, CA, USA). All values are presented as mean ± standard deviation (SD). Data are representative of at least two independent experiments analysed with duplicate or triplicate measurements (biological replicates). IC_50_ values were calculated by non-linear regression using the equation (Y = 100/(1 + 10^((LogIC_50_-X)*HillSlope)), with X = log of concentration, Y = normalised response (0–100%)). The statistical analysis of dose-response experiments was performed using two-way ANOVA with Bonferroni’s correction for multiple comparisons, to compare inhibition by Arnicae planta tota and Arnicae flos at the respective concentrations. Two-group-comparison testing was performed using an unpaired *t*-test. Data from gene expression analysis and animal experimentation were statistically analysed using one-way ANOVA followed by Dunnett’s multiple comparison test, to assess the effect of Arnica extracts relative to the vehicle control or to compare the effects of Arnicae planta tota and Arnicae flos, as indicated. A *p*-value ≤ 0.05 was considered statistically significant.

## 5. Conclusions

Arnicae planta tota showed superior anti-inflammatory activity along the NF-κB—eicosanoid inflammation pathway, compared to Arnicae flos, in a variety of *in vitro* and *in vivo* assays. Enhanced anti-inflammatory properties of Arnicae planta tota might be due to the increased and broader content in bioactive substances of the extract compared to Arnicae flos. Our data suggest that Arnicae planta tota-containing products might be more active in alleviating inflammation symptoms than those based on Arnicae flos alone.

## Data Availability

The data presented in this study are available within the article and Supplementary Materials.

## Supplementary Materials

The following supporting information can be downloaded at https://www.mdpi.com/article/10.3390/plants12061348/s1, Figure S1: Relative abundance of sesquiterpene lactone (SL) and thymol derivatives determined by HPLC in Arnicae planta tota and Arnicae flos extracts; Figure S2: Controls of NF-κB reporter assay experiment; Figure S3: Cytotoxicity control for the gene expression experiment in LPS-stimulated THP-1 cells; Figure S4: Controls for 5-LO and COX-2 enzyme inhibition assays; Figure S5: Controls for inhibition of 5-LO product and PGE2 release from isolated human peripheral blood cells.
