# Supplementary material for: Anti-Inflammatory Activities of Arnica montana Planta Tota versus Flower Extracts: Analytical, In Vitro and In Vivo Mouse Paw Oedema Model Studies
Source: Plants (Basel). 2023 Mar 16;12(6):1348. doi: 10.3390/plants12061348 (PMC10053944; doi:10.3390/plants12061348)
Supplement: Supplementary file 1 [file plants-12-01348-s001.zip › plants-2130681-supplementary.pdf]

(a)

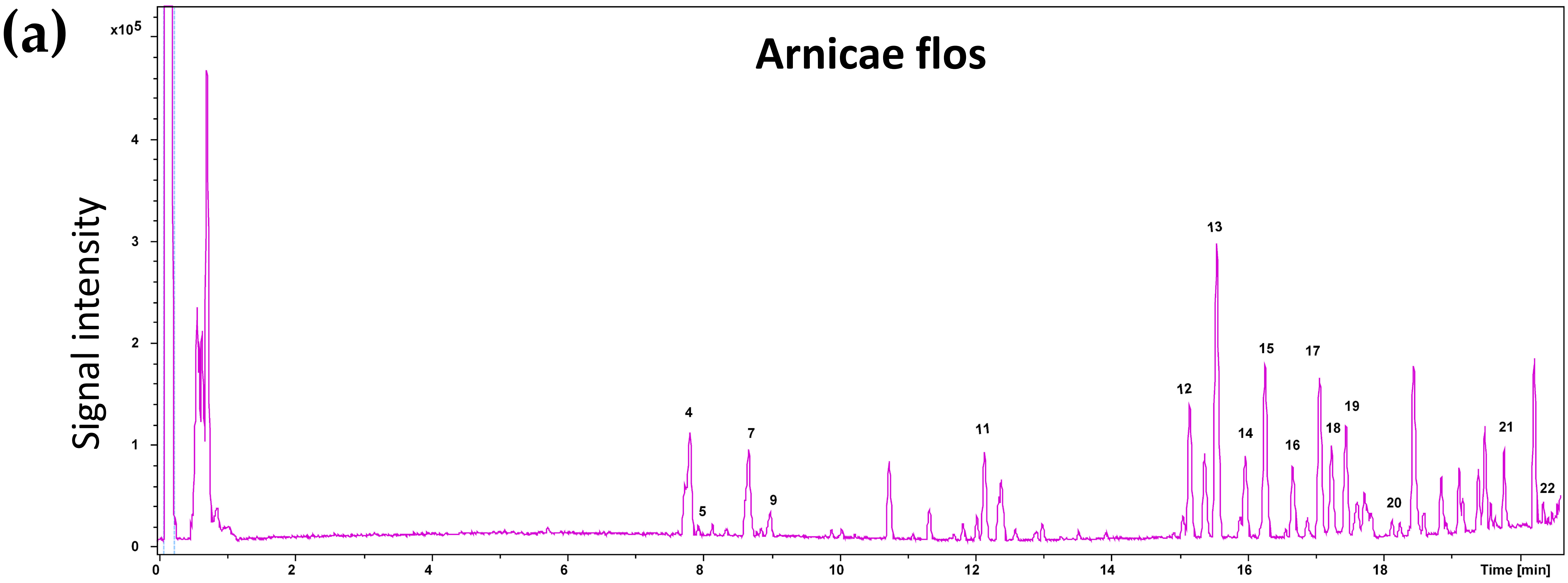

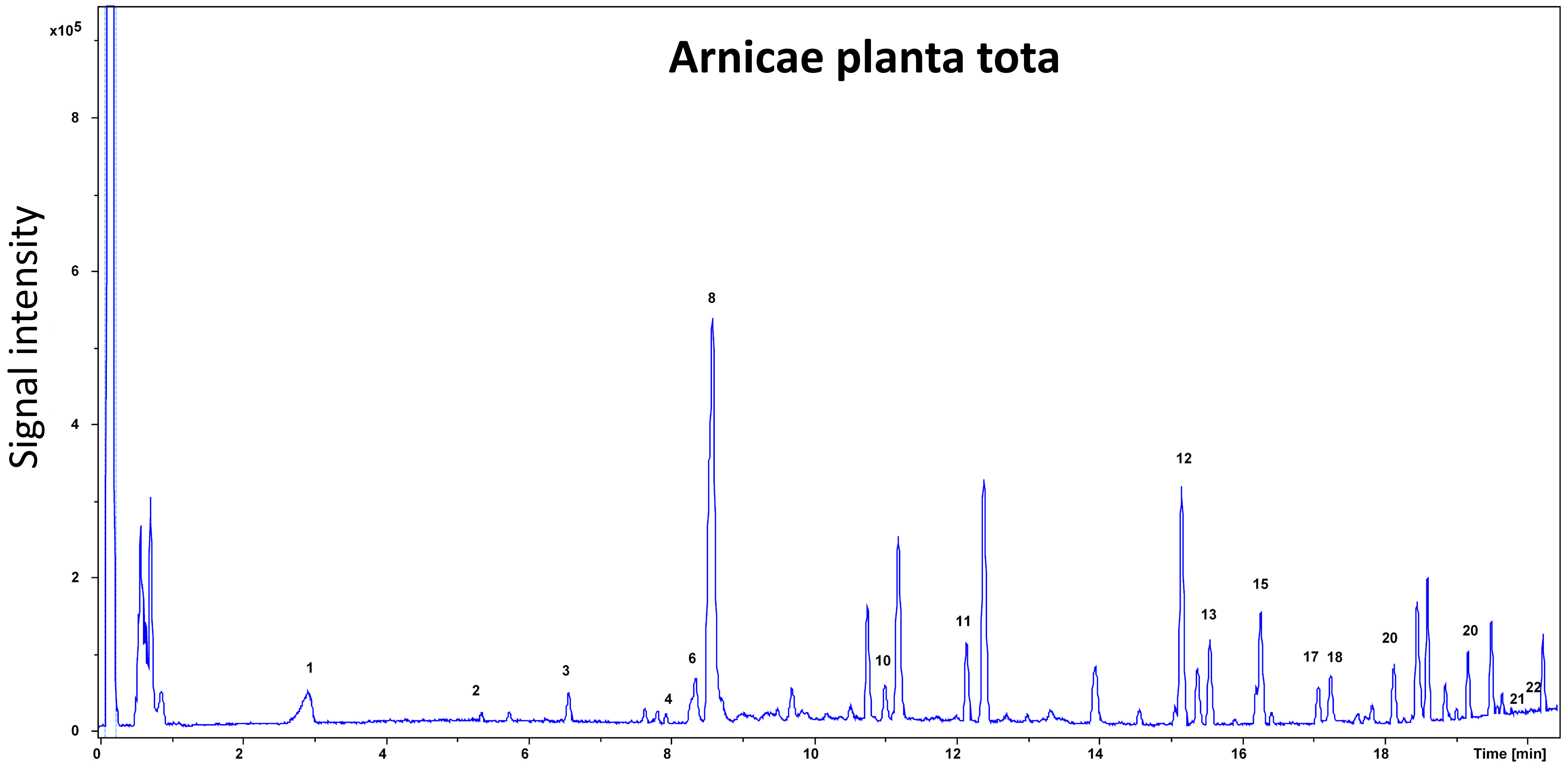

Analyte	Retention Time (min)	$[M+H]^+/[M+Na]^+$	Neutral Mass Observed/Actual	Characteristic Transition	Compound
1	3.0	377.080	354.090/354.095	163.037	Monocaffeoyl quinic acid
2	5.4	377.080	354.090/354.095	163.037	Monocaffeoyl quinic acid
3	6.6	539.114	516.124/516.126	163.037	Dicaffeoyl quinic acid
4	7.8	-	-	303.048	Quercetin derivative
5	7.9	-	-	303.034	Quercetin derivative
6	8.4	539.114	516.124/516.126	163.037	Dicaffeoyl quinic acid
7	8.7	471.086	448.096/448.100	287.053	Kaempferol glucoside
8	8.6	539.114	516.124/516.126	163.037	Dicaffeoyl quinic acid
9	8.9	501.094	478.104/478.111	317.063	Methoxy kaempferol glucoside
10	11.0	701.145	678.158/678.155	163.037	Tricaffeoyl quinic acid
11	12.1	329.135	306.145/306.146	247.131	Dihydrohelenalin acetate (DHA)
12	15.1	355.151	332.161/332.162	247.131	Dihydrohelenalin methacrylate (DHM)
13	15.6	357.166	334.176/334.178	247.131	Dihydrohelenalin isobutyrate (DHIB)
14	16.0	355.150	332.160/332.162	245.116	Helenalin isobutyrate (HIB)
15	16.3	369.166	346.176/346.178	247.131	Dihydrohelenalin tiglate (DHT)
16	16.7	367.150	344.160/344.162	245.116	Helenalin tiglate (HT)
17	17.1	371.181	348.191/348.193	247.131	Dihydrohelenalin methylbutyrate (DHMB)
18	17.2	371.181	348.191/348.193	247.131	Dihydrohelenalin isovalerate (DHIV)
19	17.4	369.166	346.176/346.178	245.116	Helenalin methylbutyrate (HMB) / Helenalin isovalerate (HIV)
20	18.1	361.159	338.170/338.172	-	8-hydroxy-9,10-diisobutyryloxythymol (HDIBT)
21	19.8	343.150	320.160/320.162	255.097	10-isobutyryloxy-8,9-epoxythymol isobutyrate (IBETI)
22	20.3	357.165	334.176/334.178	255.097	10-(2-methyl)-butyryloxy-8,9-epoxythymol isobutyrate (MBETI)

(Figure S1. see legend on next page)

(b)

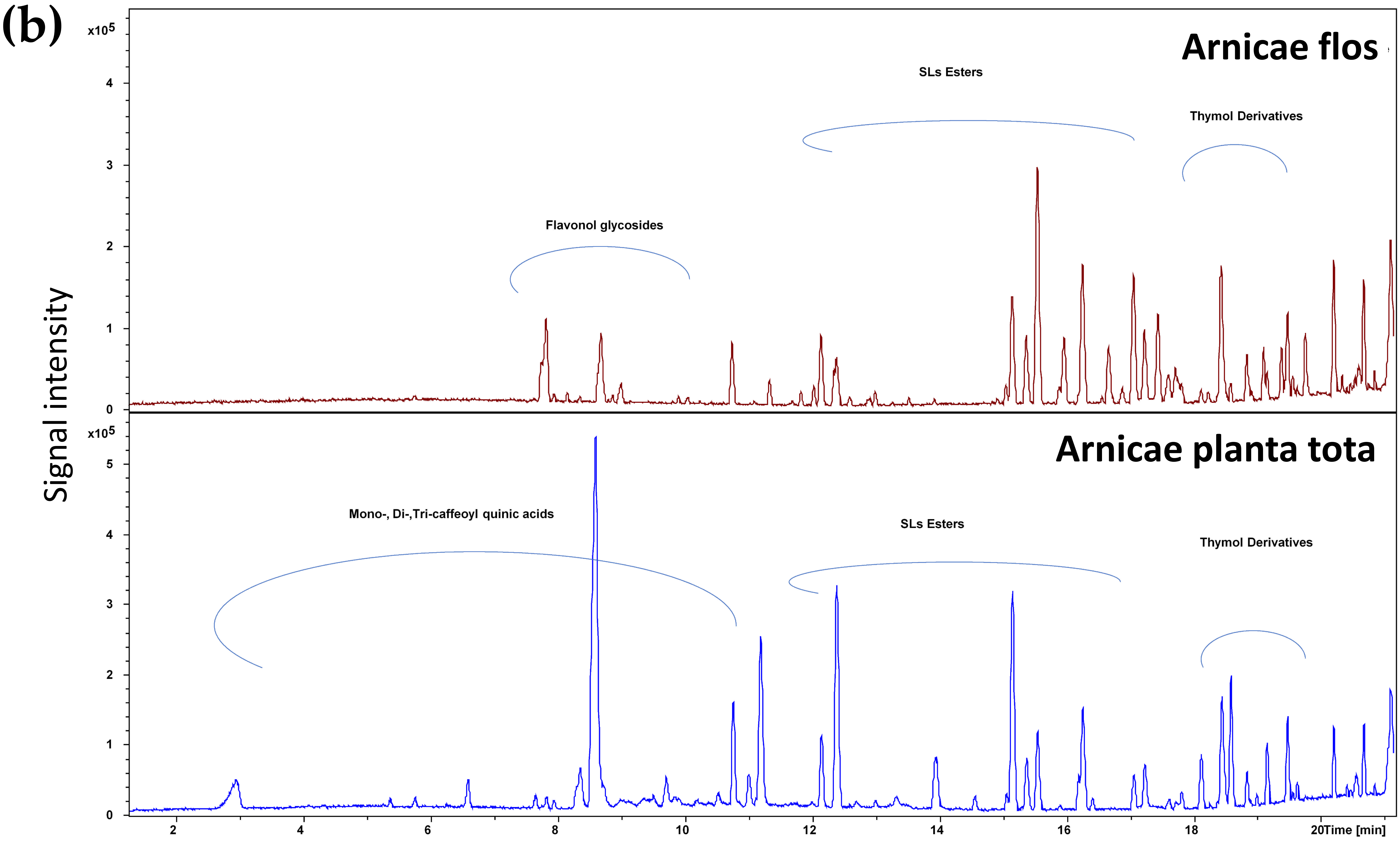

(c)

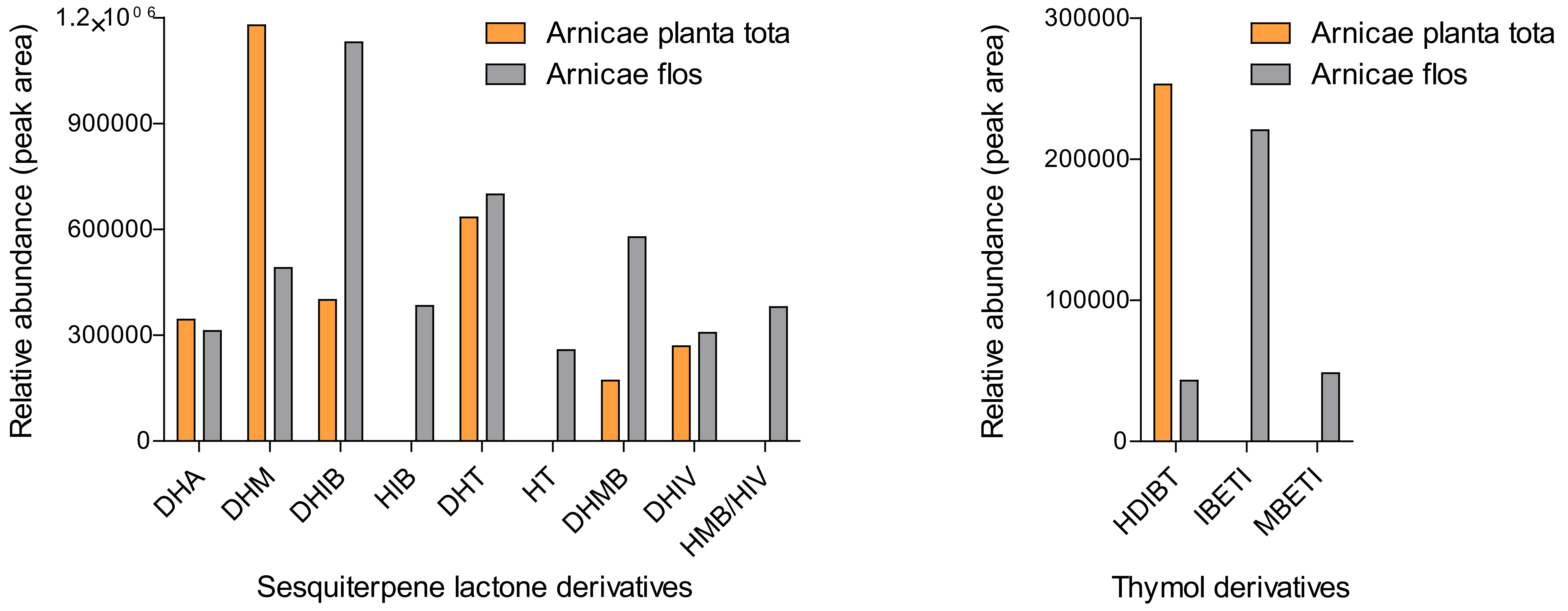

Figure S1. Relative abundance of sesquiterpene lactone (SL) and thymol derivatives determined by HPLC in Arnicae planta tota and Arnicae flos extracts. (a) Annotated spectra of UHPLC-UV-hr-qTOF/MS experiments performed on Arnicae flos and Arnicae planta tota extracts (one representative spectrum each out of three technical replicates). (**b**) Same HPLC spectra as in (a) showing the different families of identified substances. (**c**) Semi-quantitative analysis of the main sesquiterpene lactone (SL) and thymol derivatives detected by HPLC in Arnicae flos and Arnicae planta tota extracts. Abbreviations of SL derivatives: DHA, dihydrohelenalin acetate; DHM, dihydrohelenalin methacrylate; DHIB, dihydrohelenalin isobutyrate; HIB, helenalin isobutyrate; DHT, dihydrohelenalin tiglate; HT, helenalin tiglate; DHMB, dihydrohelenalin methylbutyrate; DHIV, dihydrohelenalin isovalerate; HMB, helenalin methylbutyrate; HIV, helenalin isovalerate. Abbreviations of thymol derivatives: HDIBT, 8-hydroxy-9,10-diisobutyryloxythymol; IBETI, 10-isobutyryloxy-8,9-epoxythymol isobutyrate; MBETI, 10-(2-methyl)-butyryloxy-8,9-epoxythymol isobutyrate.

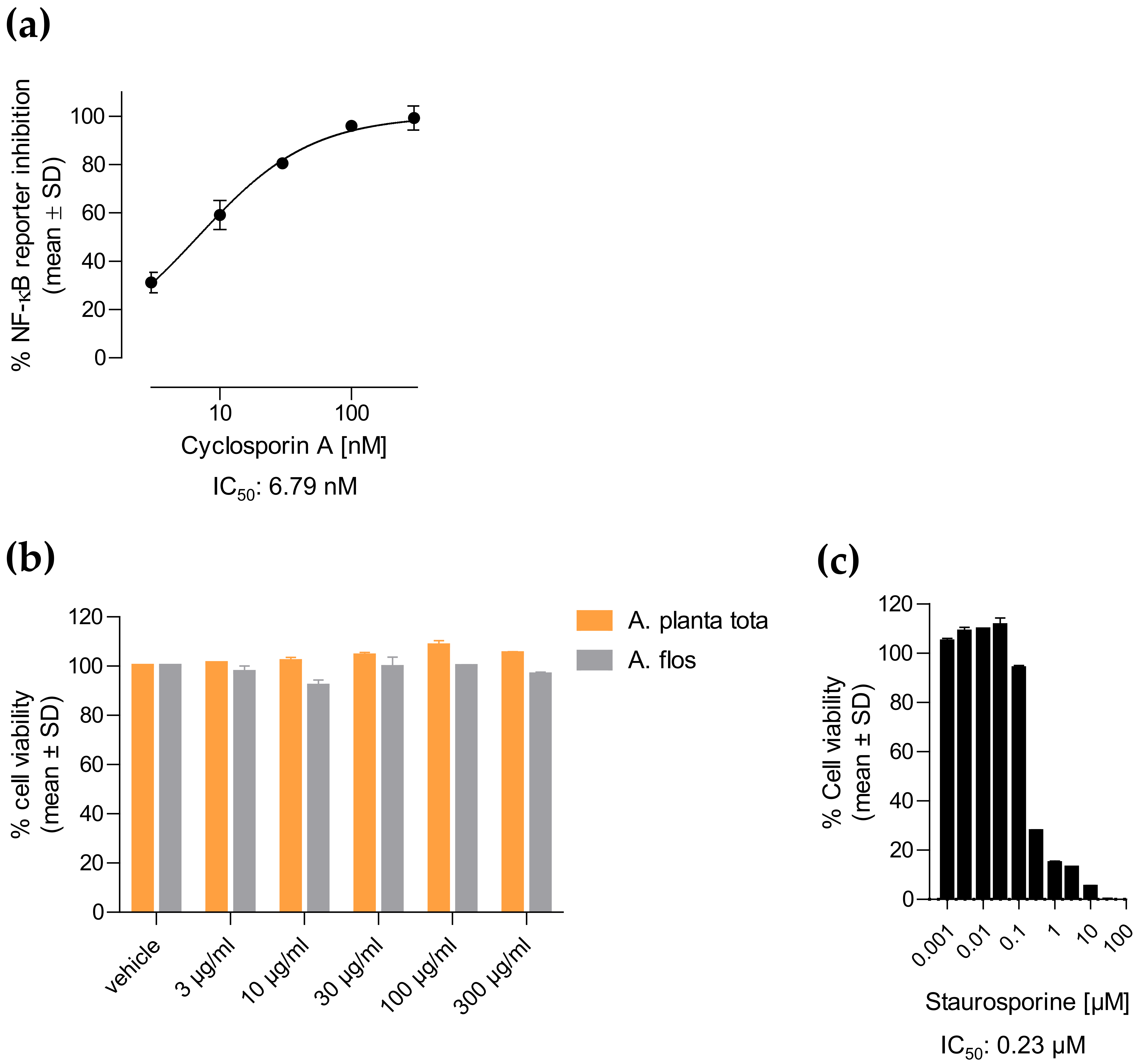

Figure S2. Controls of NF-κB reporter assay experiment shown in Figure 2. (a) Increasing concentrations (3–300 nM) of cyclosporin A served as positive control for the NF-κB reporter inhibition assay conducted in human T lymphocytic Jurkat cells. Data are expressed as the percentage of reporter inhibition relative to vehicle, and the respective half-maximal inhibitory concentrations (IC_{50}) is indicated below the graph. Data are mean ± SD of two biological replicates from one representative experiment. (**b**,**c**) Cytotoxicity assays were conducted using the CellTiter-Glo reagent under the same experimental conditions as for the NF-κB reporter assay, either in triplicate using increasing concentrations (3–300 μg/ml) of A. planta tota or A. flos (**b**), or in duplicate using increasing concentrations of staurosporine (0.001–100 μg/ml), as positive control for cytotoxicity (**c**). The percentage of cell viability was calculated relative to the vehicle control.

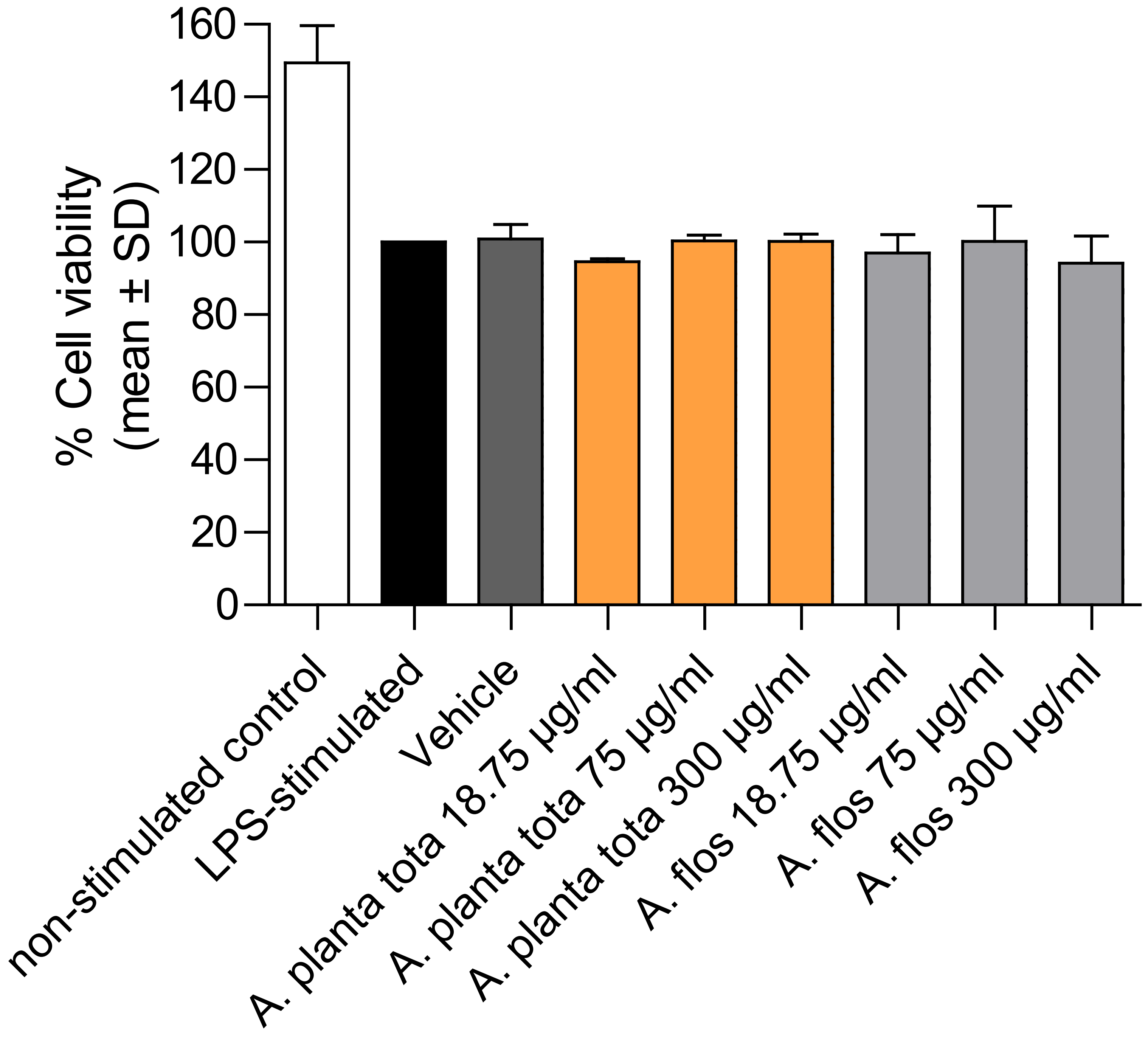

Figure S3. Cytotoxicity control for the gene expression experiment in LPS-stimulated THP-1 cells shown in Figure 3. Cytotoxicity assays were conducted in triplicate under the same experimental conditions as for the gene expression analysis, using the WST-8 reagent. The percentage of cell viability (mean $\pm$ SD of three biological replicates from one representative experiment) was calculated relative to the LPS-stimulated condition.

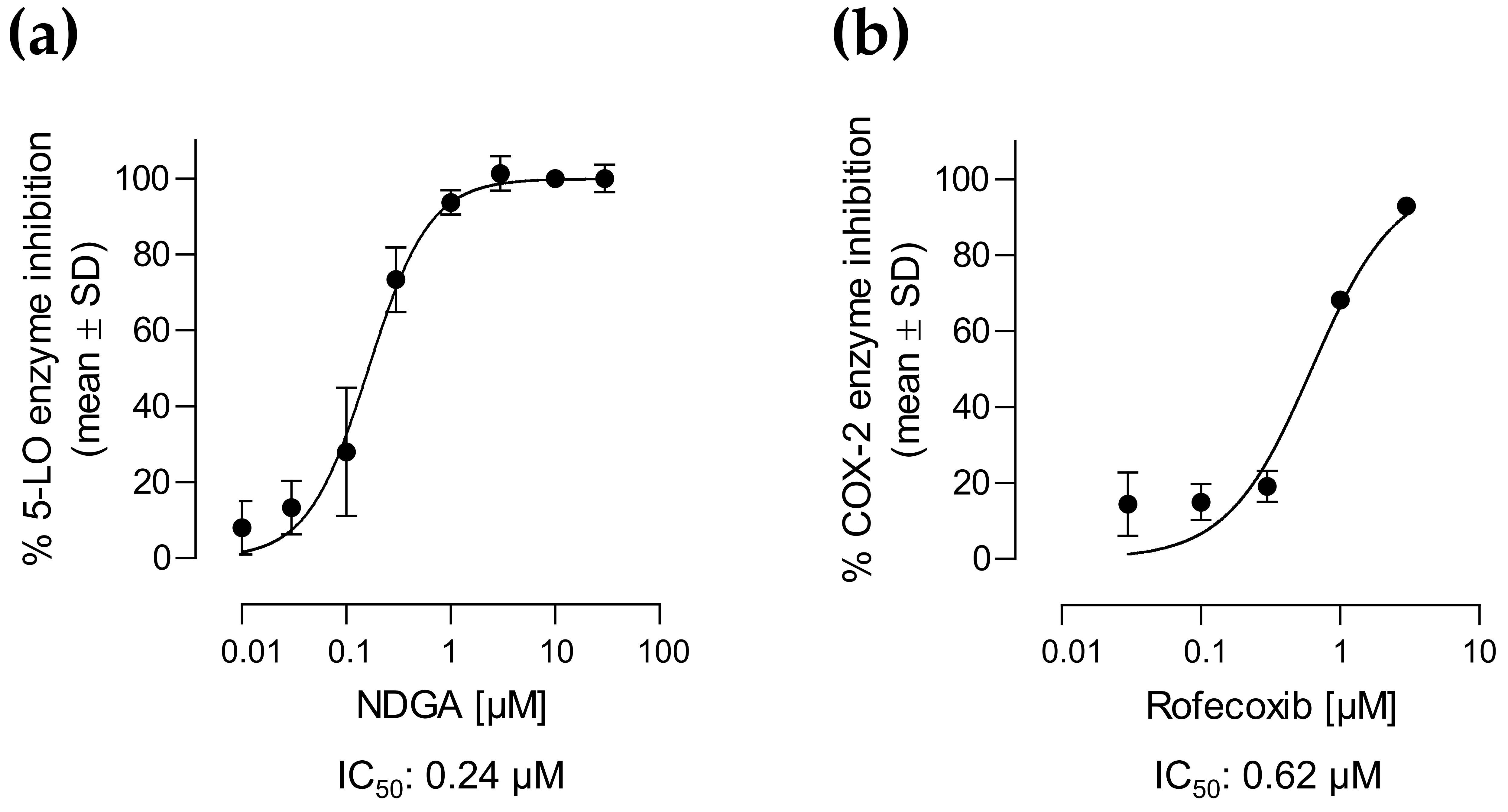

Figure S4. Controls for 5-LO and COX-2 enzyme inhibition assays shown in Figure 4. Increasing concentrations of nordihydroguaiaretic acid (NDGA; 0.01–30 µM) (**a**) and of Rofecoxib (0.03–3 µM) (**b**) served as positive controls for inhibition of 5-LO and COX-2 enzymes, respectively. Cell-free assays were conducted under the same experimental conditions as those used to test the effect of Arnica montana extracts. Data are mean ± SD of two biological replicates from one representative experiment and are expressed as the percentage of enzyme inhibition relative to vehicle. The respective IC_{50} values are indicated under each graph. IC_{50} were in the same range as those specified by the assay manufacturer (0.29 µM for NDGA and 0.42 µM for rofecoxib; Eurofins).

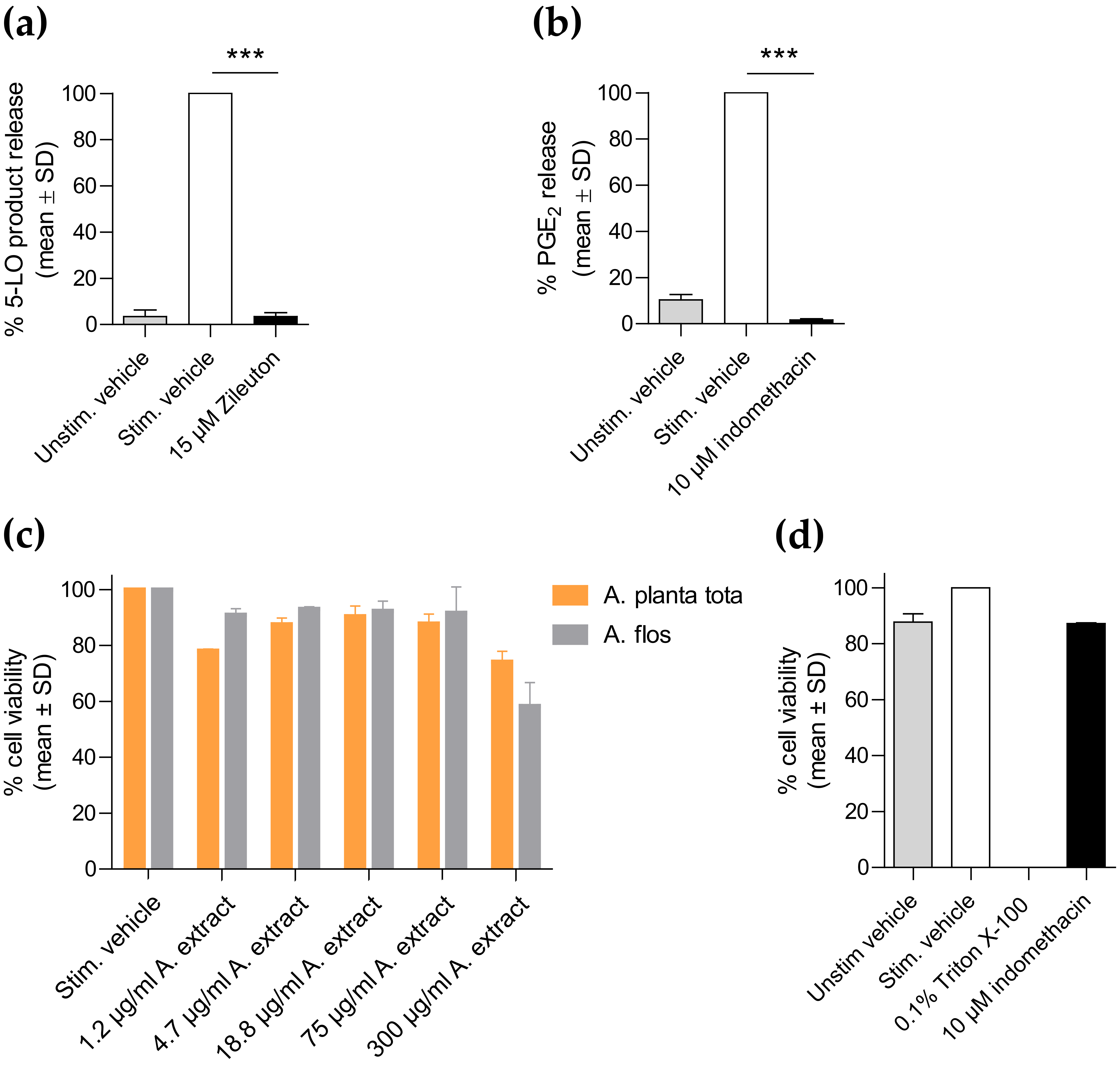

Figure S5. Controls for inhibition of 5-LO product formation and PGE_2 release from human peripheral blood cells (see Figure 5). (**a**,**b**) Zileuton (15 µM) and indomethacin (10 µM) served as positive controls for the inhibition of 5-LO and COX-2 product formation assays, respectively. Assays were conducted under the same experimental conditions as those shown in Figure 5. Data are expressed as the percentage of released products relative to the (stimulated) vehicle control. The difference in product release between vehicle and the inhibited condition (or unstimulated control) was assessed with a one-way ANOVA followed by Dunnett's multiple comparison test; only the relevant *p*-values (inhibitor *vs.* vehicle) are shown (***, $p<0.001$). (**c**,**d**) Cytotoxicity MTT assay was conducted in triplicate on isolated monocytes under the same experimental conditions as those used for the PGE_2 release assay (Figure 5b). Indomethacin (10 µM) was included as reference inhibitor of PGE_2 release and Triton X-100 served as positive control for cytotoxicity (**d**). Data are expressed as the percentage of cell viability relative to the stimulated vehicle control. Of note, no cytotoxicity control was conducted for the 5-LO product release experiment given the very short duration of the assay (25 min incubation time with Arnica montana extracts in total; see Materials and Methods).
